# Gesundheits- und Risikoverhalten und riskante Verhältnisse: Gefängnis als Herausforderung der Gesundheitsversorgung

**DOI:** 10.1007/s00103-025-04137-y

**Published:** 2025-11-03

**Authors:** Heino Stöver, Ulla-Britt Klankwarth

**Affiliations:** 1 Bundesverband für akzeptierende Drogenarbeit und humane Drogenpolitik (akzept e. V.), Südwestkorso 14, 12161 Berlin, Deutschland; 2 Frankfurt University of Applied Sciences, Frankfurt am Main, Deutschland; 3 Bundesinitiative Kinder von Inhaftierten (KvI), Treffpunkt e.V., Nürnberg, Deutschland

**Keywords:** Healthy Prisons, Äquivalenzprinzip, Gefängnismedizin, Substanzgebrauch, Gesundheitsförderung, Healthy Prisons, Equivalence of care, Prison healthcare, Substance use, Health promotion

## Abstract

Freiheitsentzug geht mit erheblichen gesundheitlichen Belastungen einher: Gefangene leiden überdurchschnittlich oft an chronischen oder psychischen Erkrankungen und Suchtproblemen. Während die Primärversorgung in den Anstalten meist regelmäßig und teils sogar schneller als außerhalb gewährleistet ist, bleibt die Versorgung komplexerer Behandlungsbedarfe häufig auf wenige Justizvollzugskrankenhäuser beschränkt. Strukturelle Defizite bestehen in der eingeschränkten freien Arztwahl, der limitierten spezialärztlichen Versorgung, der Trennung vom regulären Gesundheitssystem sowie in Versorgungslücken an den Übergängen zwischen Haft und Freiheit.

Trotz des Äquivalenzprinzips entspricht die intramurale Gesundheitsversorgung häufig nicht dem Standard der gesetzlichen Krankenversicherung (GKV). Eine Einbeziehung von Gefangenen in die GKV sowie eine Verantwortungsübertragung auf Gesundheitsministerien werden international empfohlen und könnten die Versorgung verbessern.

Besonders kritisch ist die Versorgung von Menschen mit Substanzgebrauchsstörungen: Zugang zu evidenzbasierter Therapie ist lückenhaft und regional sehr unterschiedlich. Auch Tabakentwöhnung und psychosoziale Hilfen sind unzureichend vorhanden. Auch Ernährung ist häufig unausgewogen, während Sport- und Bewegungsangebote bislang ungenutzt bleiben.

Ein interdisziplinäres Versorgungskonzept im Sinne des „Healthy-Prisons“-Ansatzes fehlt bislang weitgehend. Das baden-württembergische Modell mit Gesundheitsberichterstattung, telemedizinischen Ansätzen, altersgerechter Versorgung und Qualitätszirkeln könnte bundesweit als Vorbild dienen. Notwendig ist ein Paradigmenwechsel von der anstaltszentrierten Fürsorge hin zu partizipativer, präventiver Gesundheitsförderung, die sowohl Gefangene als auch Bedienstete einbezieht.

## Abstract (en)

Incarceration is associated with significant health burdens. Prisoners suffer disproportionately from chronic diseases, mental disorders, and substance use disorders. While primary care in prisons is usually provided regularly and sometimes even faster than outside, care for more complex treatment needs is often limited to few facilities. Structural deficits include restricted freedom of physician choice, limited specialist access, separation from the regular health system, and gaps at the transitions from incarceration to release.

Despite the principle of equivalence, prison healthcare often fails to meet the standards of the statutory health insurance (in German: Krankenversicherung [GKV]). International recommendations call for the inclusion of prisoners in the GKV as well as for a transfer of healthcare responsibility from ministries of justice to ministries of health.

Particularly challenging is the treatment of individuals with substance use disorders: access to evidence-based therapies is inconsistent and varies greatly from region to region. Smoking cessation and psychosocial support are also inadequate. Nutrition is also often unbalanced, while sports and physical activities are often inadequately implemented.

To date, a comprehensive interdisciplinary healthcare strategy aligned with the “Healthy Prisons” approach is largely absent in Germany. The Baden-Württemberg model featuring health reporting, telemedicine approaches, age-appropriate care, and quality circles, could serve as a nationwide example. A paradigm shift is required from institution-centered care toward participatory, preventive health promotion that involves both prisoners and staff.

## Einleitung

Freiheitsentzug geht mit erheblichen gesundheitlichen Belastungen einher. Die Trennung von sozialen Bezugspersonen, Isolation, Bewegungsmangel, Perspektivlosigkeit und Schuldgefühle beeinträchtigen die psychische und physische Gesundheit der Inhaftierten erheblich. Unter diesen Bedingungen ist eine qualitätsgesicherte medizinische Versorgung ein zentraler Baustein für Resozialisierung und Rückfallvermeidung [1].

Die gesundheitliche Versorgung in der Justizvollzugsanstalt (JVA) steht dabei vor wachsenden Herausforderungen. So ist eine deutliche Zunahme chronischer Erkrankungen und multimorbider Krankheitsbilder zu verzeichnen, die in Kombination mit einer steigenden Lebenserwartung altersgerechte Versorgungs- und Pflegekonzepte erforderlich macht [2]. Parallel dazu nimmt die Prävalenz somatischer Erkrankungen zu [3], ebenso wie die Zahl psychischer Auffälligkeiten unter den Inhaftierten [4]. Hinzu kommt ein wachsender Anteil nicht Deutsch sprechender Gefangener sowie eine steigende Zahl von Personen aus Herkunftsländern mit hoher Krankheitslast, darunter auch schwerwiegende Infektionserkrankungen wie Tuberkulose [5]. Besonders gravierend sind die weitverbreiteten Substanzgebrauchsstörungen, die häufig in multipler Form auftreten und eng mit infektiologischen Folgeerkrankungen wie Hepatitis, Infektionen mit dem humanen Immundefizienz-Virus (HIV) oder Acquired Immunodeficiency Syndrome (AIDS) verbunden sind [6].

Demgegenüber stehen strukturelle Einschränkungen angemessener gesundheitlicher Versorgung. Ein zentrales Problem stellt die fehlende freie Arztwahl dar. Inhaftierte sind gezwungen, sich vollständig auf die ihnen zugewiesene medizinische Fachkraft zu verlassen, was das Vertrauensverhältnis zwischen Patient und Behandelnden stark belasten kann. Gerade im Gefängniskontext ist es daher unerlässlich, dass Ärzte ihre Unabhängigkeit stets sichtbar machen [3]. Besonders im Frauenvollzug zeigt sich diese Problematik deutlich. Formuliert wird dies u. a. in der Forderung nach gendergerechter/-sensibler ärztlicher Versorgung der ’Arbeitsgemeinschaft Frauenvollzug’ [7, 8]. Ein weiteres strukturelles Defizit betrifft die Übergänge zwischen Freiheit und Haft, die regelmäßig mit Versorgungsabbrüchen und mangelhafter Anschlussversorgung einhergehen [9]. Die intramuralen (lateinisch: „intra“: hinein/innerhalb, „murus“: Mauer) medizinischen Dienste sind zudem oft nur auf die primärmedizinische Grundversorgung ausgelegt. Sekundäre und tertiäre medizinische Bedarfe – etwa bei Ko- oder Multimorbiditäten sowie psychiatrischen, geriatrischen, infektiologischen oder suchtmedizinischen Erkrankungen – werden häufig nicht ausreichend abgedeckt [10]. Gesundheitlich stark vorbelastete Gefangene, v. a. mit psychischen Störungen oder Erkrankungen, stellen enorme Herausforderungen dar, die mit keiner hausärztlichen, eher aber mit einer Schwerpunktpraxis verglichen werden können, die in der JVA allenfalls von einigen Justizvollzugskrankenhäusern (JVK) abgedeckt werden [11]. Die Erwartungen an eine spezifische Ausgestaltung der „Anstaltsmedizin“ üben sowohl von innen als auch von außen enormen Druck auf Ärzte aus. Einerseits wird verlangt, dass sie Abstinenz – d. h. den vollständigen Verzicht auf den Konsum von Alkohol oder anderen Substanzen – fördern und sicherheitsrelevante Entscheidungen mittragen. Andererseits gibt es von Teilen der Öffentlichkeit die Forderung, dass die medizinische Versorgung im Gefängnis spürbar unter dem Niveau in Freiheit liegen müsse. Solche Spannungsfelder führen zu erheblichen Interessenkonflikten im Berufsalltag [6]. Iatrogene (griechisch: „vom Arzt verursachte“) Störungen/Erkrankungen stellen besondere Herausforderungen für die medizinische Versorgung dar, da sie besonders schwer zu diagnostizieren sind. Dementsprechend sind auch die Probleme in der Versorgung und Organisation sehr umfangreich.

Der Beitrag thematisiert die gesundheitlichen Herausforderungen und strukturellen Defizite der JVA und plädiert für einen Paradigmenwechsel von einer reaktiven Gesundheits*für*sorge hin zu einer umfassenden Gesundheits*förderung* im Sinne eines „Healthy-Prison“-Ansatzes. Besonderes Augenmerk gilt dem bestehenden Parallelsystem intramuraler Gesundheitsversorgung, das in vielen Bereichen nicht dem Äquivalenzprinzip entsprechen kann.

## Von der Gesundheits*für*sorge zur Gesundheits*förderung*

Im Bundesstrafvollzugsgesetz (§ 56–66 StVollzG) wird von „Gesundheitsfürsorge“ gesprochen – ein veraltetes, überwiegend kuratives Versorgungskonzept. Präventive (u. a. Impfungen, Vorsorgeuntersuchungen) und partizipative (gefangene Menschen als Partner betrachten, die es zu stärken, zu befähigen und zu beteiligen gilt) Ansätze finden bisher kaum Anwendung. Die Begriffe „Anstaltsmedizin“ oder „Gefängnismedizin“ greifen zu kurz: Sie suggerieren eine Sonderversorgung unterhalb des Standards der gesetzlichen Krankenversicherung (GKV), obwohl das Äquivalenzprinzip gilt. Gesundheitsförderung im Strafvollzug sollte partizipativ, ressourcen- und settingorientiert gestaltet sein und alle Beteiligten – auch Bedienstete [12] – sowie die baulichen Bedingungen einbeziehen. Der Begriff „Healthy Prisons“ beschreibt dies prägnant [13]. Ein entsprechendes Versorgungskonzept fehlt jedoch in fast allen Bundesländern – mit Ausnahme Baden-Württembergs.

Obwohl Begriffe wie Prophylaxe, Prävention und Resozialisierung häufig fallen, bleiben die tatsächlichen Möglichkeiten angesichts eingeschränkter Angebote und hoher Problemkonzentration – etwa bei Sucht oder psychischen Erkrankungen – begrenzt. Die folgenden Abschnitte analysieren die Problemlage und zeigen Optimierungspotenziale auf.

## Parallelsystem intramuraler Gesundheitsversorgung

Anders als in vielen anderen europäischen Ländern [14] wird in Deutschland weiterhin auf das Modell der Anstalts- und Vertragsärzte gesetzt. In anderen Staaten übernehmen hingegen kommunale Gesundheitsdienste oder Hausärzte die Versorgung von Gefangenen. Obwohl der Gesetzgeber in den 1970er-Jahren die Einbeziehung in die GKV beabsichtigte, wurde dieser Weg nicht weiterverfolgt. Lediglich einige Bundesländer lockern die spezialärztliche Versorgung in JVK zugunsten gesicherter Krankenräume in öffentlichen Kliniken [11]. Angesichts der erheblichen gesundheitlichen Probleme in Haft stellt sich die Frage, ob das geltende Parallelsystem überhaupt den internationalen und nationalen Standards des Äquivalenzprinzips gerecht werden kann. Kernprobleme dieses Parallelsystems bleiben:

- das Herausfallen der Gefangenen aus der GKV (mit erheblichen Problemen des Zeitverzugs, wieder in die Krankenkasse aufgenommen zu werden [15]),
- der Wegfall der freien Arztwahl und die damit verbundenen Probleme des Misstrauens gegenüber dem Einhalten der ärztlichen Schweigepflicht und der Vertraulichkeit beim Umgang mit sensiblen Daten
- sowie der erhebliche Zeitverzug in der Einführung von State-of-the-Art-Medizin in der JVA, die oft erst durch richterliche Entscheidungen erzwungen wird.^1^

„Anstaltsmedizin ist Monopolmedizin“ [16], dieser Umstand verlangt von den beteiligten Akteuren ein hohes Maß an Selbstreflexion im Sinne der Frage: „Wie gehe ich mit der mir zugewiesenen Macht um, wie bemühe ich mich um die Einhaltung von GKV-Standards, wieweit trenne ich mich von einer traditionellen Anstaltsmedizin in Richtung gesundheitlicher Versorgung Gefangener?“ [16].

## Einbeziehung Gefangener in die Kranken- und Pflegeversicherung

Bis heute unterliegen Strafgefangene und Sicherungsverwahrte in Deutschland einer gesetzlichen Arbeitspflicht, genießen dabei jedoch keinen umfassenden sozialrechtlichen Schutz. Zwar sind sie in die Unfall- und Arbeitslosenversicherung einbezogen (§ 2 Abs. 2 S. 2 Sozialgesetzbuch (SGB) VII sowie § 26 Abs. 1 Nr. 4 SGB III), jedoch nur ein kleiner Teil in die Kranken‑, Pflege- und Rentenversicherung. Bereits mit dem StVollzG wurde vor fast vier Jahrzehnten eine Einbeziehung in die Sozialversicherungen angekündigt. Damals wie heute gilt, dass es „nicht gerechtfertigt ist, neben den notwendigen Einschränkungen, die der Freiheitsentzug unvermeidbar mit sich bringt, weitere vermeidbare wirtschaftliche Einbußen zuzufügen“ [17]. Doch das damals in den §§ 190 ff. StVollzG angekündigte besondere Bundesgesetz, mit dem die Gefangenen in die Sozialversicherungen einbezogen werden sollten, ist bis heute nicht erlassen worden [18]. Um soziale Gerechtigkeit herzustellen, müssen Strafgefangene und Sicherungsverwahrte vollständig in die gesetzliche Kranken- und Pflegeversicherung aufgenommen werden. Die gegenwärtige Ungleichbehandlung gegenüber Berufsfreigängern ist mit Art. 3 des Grundgesetzes kaum vereinbar. Zudem wirkt sich die fehlende Versicherungspflicht nachteilig auf Ansprüche nach der Haftentlassung aus, etwa auf Leistungen der Pflegeversicherung, die an Mindestversicherungszeiten geknüpft sind.

## Übertragung der Verantwortlichkeit der gesundheitlichen Versorgung vom Justizministerium auf die Gesundheitsministerien [14]

Die internationale Diskussion zur Qualität der gesundheitlichen Versorgung in Haft wird seit 1995 maßgeblich durch das *Health in Prisons Programme* (HIPP) der Weltgesundheitsorganisation (WHO) geprägt. Es zielt auf die Verbesserung von Haftbedingungen, Gesundheit und eine engere Verzahnung der Gefängnismedizin mit den nationalen Gesundheitssystemen [19]. Gesundheitsdaten der europäischen Mitgliedsorganisationen fließen in die Datenbank *Health In Prisons European Database* (HIPED) ein [19–21]. Die WHO empfahl in der *Declaration on Prison Health as Part of Public Health* (Moscow Declaration) 2003, Gesundheitsministerien stärker in die Versorgung in Haft einzubinden [22]. Der Europarat griff diese Empfehlung in den *European Prison Rules* auf und forderte in Recommendation R(98)7 eine klare Aufgabenverteilung zwischen Justiz- und Gesundheitsministerien zur Umsetzung integrierter Gesundheitspolitik: „The role of the Ministry responsible for health should be strengthened in the domain of quality assessment of hygiene, healthcare and organizations of health services in custody, in accordance with national legislation. A clear division of responsibilities and authority should be established between the Ministry responsible for health or other competent ministries, which should co-operate in implementing an integrated health policy in prison“ [23].

Erfahrungen aus Ländern wie Italien, Finnland, Spanien (Baskenland) oder dem Vereinigten Königreich zeigen, dass eine Umstellung die Versorgungsqualität, Transparenz, Ausbildung des Personals und den Übergang in die Freiheit deutlich verbessert und Gefangene stärker in Gesundheitsförderung einbindet [14].

## Einbezug in die Gesundheitsberichterstattung

Um zunächst den Umfang und die Qualität der gesundheitlichen Versorgung in Haft bemessen zu können, sollte der Ist-Zustand detailliert beschrieben und analysiert werden. Als einziges Bundesland werden in Baden-Württemberg seit dem Jahr 2008 systematisch Gesundheitsdaten der Gefangenen in den JVAen erhoben und durch das Ministerium der Justiz und für Migration Baden-Württemberg (JuM BW) in einem jährlichen Gesundheitsbericht zusammengefasst, ausgewertet und als Planungsgrundlage genutzt [24]. Der Bericht gliedert sich in folgende Rubriken:

- Justizvollzugsstatistik,
- Infektionskrankheiten,
- Gebrauch von Drogensubstanzen,
- psychische Störungen,
- Prävention,
- Betreuung,
- Risikoverhalten im Vollzug,
- Krankenhausaufenthalte,
- Vorstellungen bei Fachärzten und Psychotherapeuten,
- zahnärztliche Versorgung.

Diese Daten bilden die Grundlage für eine Steuerung und Mittelallokation der gesundheitlichen Versorgung Gefangener, der Arbeitsbedingungen Bediensteter und der räumlich-physikalischen Bedingungen [24].

## Risikoverhalten Gefangener und strukturelle Probleme als Herausforderungen für Haftanstalten

Ein umfassendes Gesundheitsversorgungskonzept, das auf eine Verbesserung der Lebens- und Arbeitsbedingungen Gefangener sowie Beschäftigter im Sinne eines Healthy-Prisons-Ansatzes abzielt, gibt es – mit Ausnahme ansatzweise in Baden-Württemberg – in keinem Bundesland. Dabei wären angesichts der massiven gesundheitlichen Herausforderungen umfassende, interdisziplinär anzugehende Konzepte von großer Bedeutung: verbreitetes Risikoverhalten in Bezug auf Substanzkonsum (v. a. Opioide, neue psychoaktive Substanzen (NPS), Tabak, polyvalenter Drogenkonsum) einerseits, u. a. strukturell bedingte Bewegungsarmut und z. T. Ernährungsprobleme andererseits.

### Substanzgebrauchsstörungen

Konsumierende von legalen und illegalen Drogen bilden im Vollzug eine große und sowohl gesundheitlich als auch sozial besonders gefährdete Gruppe [6]. Etwa 30–40 % der in Deutschland Inhaftierten sind von Drogenmissbrauch und -abhängigkeit betroffen [25]. Damit verbunden sind für die Betroffenen gesundheitliche Folgeprobleme, v. a. Drogenkonsumstörungen, -notfälle, virale Infektionskrankheiten (v. a. HIV/Hepatitiden), Komorbiditäten (chronisch obstruktive Lungenerkrankungen) und weitere gesundheitliche und soziale Notlagen: Beschaffungsdruck im Gefängnis mit neuen Abhängigkeiten, neuen Drogen (z. B. NPS), Risiken durch verunreinigte Stoffe, Gewalt, Verschuldungen etc.

Obwohl in den letzten 30 Jahren in Freiheit bedeutende Fortschritte in der Suchtmedizin und bei psychosozialen Interventionen erzielt wurden, spiegeln sich diese Entwicklungen im Strafvollzug trotz der weiterhin weitverbreiteten abhängigen und missbräuchlichen Konsummuster nicht im gleichen Maße wider. Im Gegensatz zur erheblichen Diversifizierung des Hilfesystems außerhalb der JVA wird dort nach wie vor hauptsächlich auf das zentrale Ziel der Abstinenz gesetzt. Vor allem der Zugang zu erprobten und anerkannten Hilfe- und Behandlungsmöglichkeiten während der Haft ist in einigen Bereichen der Suchtkrankenversorgung im Vergleich zur Versorgung in Freiheit deutlich unzureichend (v. a. Pharmakotherapie, Infektionsprophylaxe, Rauch- und Alkoholentwöhnung). Evidenzbasierte Kernstrategien zur Behandlung der Opioidabhängigkeit, wie etwa die Substitutionsbehandlung, werden in vielen Haftanstalten oft erst nach langem Zeitverzug eingeführt, sind nicht flächendeckend verfügbar oder fehlen in einigen Bundesländern sogar vollständig [26]. Dies verursacht Behandlungsabbrüche, die sich teils deutlich negativ auf die Gesundheit in und nach der Inhaftierung auswirken.

Alkoholkonsumstörungen (mit z. T. lebensgefährlichen Alkoholentzugserscheinungen mit deliranten Zustandsbildern und zerebralen Krampfanfällen) treten v. a. gehäuft bei Gefangenen mit Ersatzfreiheitsstrafe auf. Eine leitliniengerechte Versorgung [27] sieht eine engmaschige klinische Kontrolle und Behandlung vor.

Da der polyvalente Drogenkonsum bei Gefangenen weitverbreitet ist, werden Medikamentenentzüge (v. a. durch Benzodiazepine verursachte Konsumstörungen, v. a. Pregabalin [28]) häufig nötig [29].

Eine entschlossene Entkriminalisierungspolitik, einschließlich einer drastischen Reduktion der Ersatzfreiheitsstrafen, würde die Zahl der drogenkonsumierenden Gefangenen massiv senken. Dies würde die Behandlungsbedingungen für die verbleibenden drogenkonsumierenden Gefangenen und die Arbeitsbedingungen für die Bediensteten erheblich verbessern.

Eine adäquate Unterstützung für die umfangreiche Gruppe drogenkonsumierender Gefangener setzt ein umfassendes und differenziertes Hilfekonzept voraus, das Menschen mit Substanzgebrauchsstörungen (einschließlich psychischer, somatischer und infektiologischer Begleiterkrankungen) in jeder JVA berücksichtigt, mit allen Abteilungen abgestimmt ist und eng mit weiterführenden Hilfsangeboten in der (Heimat‑)Kommune sowie der Selbsthilfe vernetzt wird. Dies bedeutet, dass sowohl abstinenzbezogene Hilfen als auch Harm-Reduction-Unterstützungen angeboten werden müssen [9]. Ein Drogenhilfe- und Infektionsschutzkonzept sollte sowohl auf wissenschaftlich fundierten Erkenntnissen als auch auf systematisierten Praxiserfahrungen basieren und Behandlungs- sowie Präventionsangebote für Drogenabhängigkeit und -missbrauch sowie Maßnahmen zur Infektionsprophylaxe umfassen, die sich außerhalb des Vollzugs als wirksam und effizient bewährt haben. Ähnlich wie in der Freiheit könnten durch Patientenschulungen, Peer Support/Education und Psychoedukation die Kompetenzen der Betroffenen stärker in Beratung und Behandlung einbezogen werden [9].

Der Drogenkonsum im Gefängnis unterscheidet sich für viele Inhaftierte von dem in Freiheit. Einige Gefangene konsumieren ähnlich wie in Freiheit weiter, während andere ihre bevorzugten Substanzen, Konsumformen oder die Häufigkeit des Konsums verändern. Wieder andere entscheiden sich dafür, während der Haftzeit ganz auf Konsum zu verzichten. Neben Unterschieden zwischen den einzelnen JVAen sind Engpässe bei bestimmten Substanzen häufig und das Drogenangebot ist insgesamt begrenzter und teurer als außerhalb der Haft. In Haft ist es zudem (noch) schwieriger, die Kontrolle über Art und Qualität der konsumierten Substanzen zu behalten, als in Freiheit. Aufgrund des Suchtdrucks kommt es oft zu Mischkonsum oder zum Konsum nicht bevorzugter Substanzen (wie verschiedene Medikamente, NPS oder „härtere“ Drogen als zuvor in Freiheit), wobei häufig ein eingeschränkter Zugang zu sauberen Konsumutensilien besteht. Das Risiko wird dabei nur teilweise durch alternative Konsumformen, wie bspw. Inhalation statt Injektion, reduziert. Für einige Drogenkonsumierende bietet der Strafvollzug eine als nützlich erlebte Unterbrechung des Konsums und des Lebens in der Drogenszene. Die Haftzeit wird als Chance zur gesundheitlichen Stabilisierung und Regeneration genutzt, soziale Kontakte werden gepflegt und es erfolgt eine Wiederaufnahme von Kontakten zu Angehörigen. Das Gefängnisumfeld kann somit zu einer Reduktion oder (vorübergehenden) Aufgabe des Konsums führen [30]. Eine Gruppe von Gefangenen wiederum beginnt erst in der Haft, Drogen zu konsumieren [31].

#### Opioidgebrauchsstörungen

Wie in der Freiheit so auch in der Haft stellt die Opioidagonisttherapie (oder auch *Substitutionsbehandlung*) für viele Opioidabhängige die einzige Möglichkeit dar, einen Weg aus ihrer Abhängigkeit zu finden, weniger/keine psychotropen Substanzen mehr zu konsumieren und sich gesundheitlich und sozial so zu stabilisieren, dass sie mit ihrer Abhängigkeit unter menschenwürdigen Umständen leben können. Für viele opioidabhängige Gefangene bildet die Substitutionsbehandlung die Basis für weiterführende Therapieangebote [15].

Ein zentraler Wirkmechanismus der Substitutionsmedikamente besteht darin, das Verlangen nach Opioiden, auch „Heroinhunger“ genannt, deutlich zu reduzieren. Durch die Substitutionsbehandlung opioidabhängiger Gefangener kann der fortgesetzte Opioidkonsum innerhalb der JVA nachhaltig verringert werden. Dies ist für die Inhaftierten besonders wichtig, da sie dadurch nicht mehr ständig nach Wegen suchen müssen, Drogen zu beschaffen oder zu finanzieren – Faktoren, die den Therapieprozess sonst immer wieder behindern und unterbrechen. Gleichzeitig spielt die Substitutionsbehandlung auch für das Justizvollzugssystem eine bedeutende Rolle, da sie die Dynamiken einer weitverbreiteten Sucht im Gefängnis entschärft: Tätigkeiten wie Kaufen, Tauschen, Finanzieren, Konsumieren, Erpressen und Gewalt gehören häufig zum Alltag und werden durch die Behandlung deutlich reduziert [15].

Zahlreiche Studien belegen die Wirksamkeit der Substitutionsbehandlung auch im Haftkontext und die rechtliche Bewertung in der Betäubungsmittelverschreibungsverordnung (BtMVV) ist eindeutig positiv [26]. Die Auswahl an Medikamenten ist größer und die Applikationsformen sind vielfältiger geworden [1]. Dennoch erhält bundesweit weniger als die Hälfte der opioidabhängigen Gefangenen, im Gegensatz zur Versorgungssituation außerhalb der Haft, eine solche Behandlung. Dabei zeigen sich große regionale Unterschiede. Außerdem variiert die Organisation der Substitutionsbehandlung aktuell stark von einer JVA zur nächsten [32].

Auch im Maßregelvollzug wird die Substitutionsbehandlung nur „zurückhaltend“ eingesetzt – auch dort bleibt sie sozusagen unter ihren Möglichkeiten [33–35].

#### Tabakgebrauchsstörungen

Laut der Studie von Stöver et al. [36] sind 79 % der Gefangenen in Deutschland Raucher, meist wird in den Zellen geraucht. 56 % geben an, dass ihr Tabakkonsum durch die Haft gestiegen ist. Jeder zweite Raucher hat bereits einen Entwöhnungsversuch unternommen, doch entsprechende Angebote sind selten. 76,3 % der Gefangenen, darunter viele Nichtraucher, sind Passivrauch ausgesetzt, besonders in Zellen, Freizeiträumen und Arbeitsstätten. Während Nichtraucher mehrheitlich ein Rauchverbot befürworten, akzeptieren Raucher dies meist nur, wenn ihre Hafträume ausgenommen sind.

Die hohe Raucherrate und das Interesse an Reduktion stehen im Gegensatz zu den wenigen Hilfsangeboten. Daher sind ein besserer Nichtraucherschutz sowie Maßnahmen zur Prävention und Behandlung der Nikotinsucht dringend nötig [36].

Unterstützende Entwöhnungsstrategien und Hilfsmittel fehlen in den meisten JVAen fast vollständig [37, 38]. Eine Ausnahme bildet die JVA Hameln. Die von Juvet und Niggemann [39] dort im Jahr 2024 durchgeführte Studie liefert dabei folgende Empfehlungen für die Praxis:

- Gesundheitsaufklärung und Fortbildungen,
- individuelle Unterstützungsangebote zum Rauchstopp oder zur Reduzierung des Rauchkonsums, sowohl für Gefangene als auch für Mitarbeitende,
- Aufbau von Netzwerken,
- Monitoring und Evaluation.

Prävention, Beratung und Behandlung sollten sowohl Verhalten als auch Umfeld einbeziehen und geschlechts-, alters- sowie kulturspezifische Besonderheiten sowie alltagsnahe, kostengünstige Angebote berücksichtigen. Trotz hoher Tabakkonsumprävalenz bei Gefangenen gibt es viele Ansatzpunkte für Maßnahmen zur Rauchreduktion oder -beendigung. Die breite Akzeptanz des Nichtraucherschutzes bei Gefangenen und Bediensteten ermöglicht bauliche, strukturelle und individuelle Unterstützungen (Nichtraucherhafträume, kostenlose Nikotinersatzprodukte, Gruppenangebote, Nichtrauchercoaching, Peer-Support-Unterstützungsangebote). Dabei sollten sowohl Gefangene als auch Bedienstete berücksichtigt werden, da sie stark rauchbelasteter Luft ausgesetzt sind. Die Förderung des Nichtrauchens bei Bediensteten sollte Teil der betrieblichen Gesundheitsförderung sein. Viele Gefangene wollen aus gesundheitlichen oder wirtschaftlichen Gründen aufhören, erhalten jedoch oft unzureichende Unterstützung. Passgenaue Angebote können diesen Wunsch stärken [36].

#### Psychosoziale Behandlungen von Menschen mit Substanzgebrauchsstörungen

Die Beratung und Behandlung von Menschen mit Substanzgebrauchsstörungen hat im Anstaltsalltag nachrangige Bedeutung gegenüber ordnungsbezogenen Zielen. Die Grundhaltung ist meist abstinenzorientiert und kontrollbasiert. Zielsetzungen wie „Verfestigung der Sucht entgegenwirken“ und „Abstinenz stabilisieren“ dominieren. Schadensminimierende Maßnahmen werden dagegen selten angeboten. Psychosoziale Angebote fokussieren primär auf Abstinenzmotivation, teils aber auch auf Rückfallprophylaxe und Konsumkompetenz. Im Mittelpunkt stehen Anamnese, individuelle Hilfeplanung und Weitervermittlung in Therapien [40].

Zentrale Behandlungsform bleibt die Überleitung in externe Einrichtungen nach dem Modell „Therapie statt Strafe“ (§§ 35 ff. BtMG; [41, 42]). In der Praxis wird dies jedoch häufig durch enge Auslegung des Kausalzusammenhangs zwischen Tat und Abhängigkeit blockiert [43]. Therapievorbereitende Gruppen existieren in den meisten JVAen.

Drogenfreie Stationen ermöglichen ein Leben ohne Konsum unter erhöhter Kontrolle (z. B. Urinkontrollen) und bieten teils begleitende Therapieangebote. Sie sind meist mit Privilegien wie mehr Freizeit oder Lockerungen verbunden, ihre Wirksamkeit bleibt jedoch umstritten. Im Praxisansatz des „Contingency Management“^2^ werden positive Verhaltensweisen durch Vergünstigungen (z. B. Besuche, Pakete) belohnt.

Manche sozialtherapeutischen Abteilungen bieten jungen Konsumierenden, für die eine externe Therapie nicht infrage kommt, eine interne Behandlung an – etwa nach dem *Crailsheimer Modell *[45], das fünf Schwerpunkte setzt:

- Arbeit an der Drogenproblematik,
- Heranführen an Arbeit und soziale Pflichten,
- körperliches Aufbautraining,
- Nachreifung der Persönlichkeit und
- Entlassungsvorbereitung.

## Sport und Bewegung

Im geschlossenen Strafvollzug spielt Sport eine zentrale Rolle angesichts der Bewegungsarmut von Gefangenen. (Freizeit‑)Sport hilft, die Monotonie des Gefängnisalltags zu durchbrechen, Stress abzubauen und soziale Isolation zu reduzieren [46]. Zudem ermöglicht Gefängnissport Gefangenen, sich als selbstbestimmte Subjekte mit hoher Selbstwirksamkeit zu erleben und damit aus einer hafttypischen Passivität herauszulösen. Diese Aspekte sind wesentlich für eine gelingende Resozialisierung. Welche Sportarten allerdings für welche Gefangenen zur Resozialisierung beitragen, ist jedoch unklar [47, 48].

Im Gegensatz zum Freizeitsport versteht man unter „Behandlungssport“ [49] gelenkte, sportbezogene Maßnahmen, die darauf abzielen, individuelle Verhaltensweisen der Gefangenen zu verändern. Dadurch soll der gesetzliche Behandlungs- und Erziehungsauftrag erfüllt werden, mit dem Ziel der Resozialisierung [50, 51]. „Behandlungssport hat seine Aufgaben nicht in der Vermittlung sportmotorischer Fertigkeiten, sondern ausschließlich darin, einen Beitrag zur sozialtherapeutischen Behandlung der Klienten zu leisten. Bewegung, Spiel und Sport sind nicht das Ziel dieser Einflussnahme, sondern lediglich Gegenstand, Medium oder Hilfsmittel“ [49].

Solche Sportangebote könnten beispielsweise darauf abzielen, Kooperations- und Teamfähigkeit innerhalb der Gruppe zu fördern, den Umgang mit Aggression und Frustration zu verbessern oder die Selbstwirksamkeit zu stärken. Rund ein Drittel der männlichen Gefangenen in deutschen JVAen nutzt regelmäßig die angebotenen Freizeit- und Behandlungssportaktivitäten. Dabei zeigen sich klare Präferenzen für Sportarten wie Fußball, Handball, Tischtennis, Badminton, Volleyball sowie Krafttraining und Laufen [50, 52]. Der Kraftsport spielt im Männervollzug dabei eine besondere Rolle in der Gestaltung von Männlichkeit: „Prison is an ultramasculine world where nobody talks about masculinity“ [53]. Allzu viel mehr – v. a. für Frauen und Bewegung und Sport – wissen wir allerdings über den Gefängnissport in Deutschland bislang aus Mangel an entsprechenden Studien nicht.

## Ernährung

Die Ernährung in JVAen muss besonders hohen Ansprüchen genügen, da die Insassen keine Auswahl haben und die Kost oft jahrelang verzehren. Im Gefängnis als totaler Institution ist eine ausgewogene Ernährung wichtig, da Bewegungsmangel, unausgewogene Kost, Übergewicht und Fettleibigkeit eng zusammenhängen und nach der Entlassung auch die öffentliche Gesundheit durch Folgekosten betreffen. Die Verpflegung darf kein zusätzliches Strafübel sein (OLG Zweibrücken ZfStrVo 1994, 52 = StV 1993, 488) und orientiert sich an Standards aus Krankenhäusern, Altersheimen, Bundeswehr oder Werkskantinen, basierend auf den Referenzwerten der Deutschen Gesellschaft für Ernährung (DGE; OLG Hamm NStZ 1995, 616). Seit 2018 wird mehr Obst, Salat und Gemüse und weniger Fleisch angeboten; für religiöse Vorgaben gilt „Normalkost ohne Schwein“.

Studien zur JVA-Ernährung sind selten; eine Untersuchung von Krawinkel und Halacz im Jahr 2006 [54] zeigte einen höheren Anteil an Fleisch, Fett und Brot, aber weniger Obst als in der Allgemeinbevölkerung. Empfehlungen zielen auf mehr frisches Obst, mageres Fleisch und weniger Fett ab. Insgesamt hat sich die Ernährung seit 1988 verbessert, orientiert an den D‑A-CH-Referenzwerten für die Nährstoffzufuhr. Die Verpflegung im Strafvollzug unterliegt zahlreichen Regeln und Kontrollen, die der Mahlzeit als sozialer Institution wenig Raum lassen [55]. In den meisten Gefängnissen wird das Essen für Gefangene in der Anstaltsküche portionsgerecht zusammengestellt und von einem Wagen von Haftraumtür zu Haftraumtür ausgegeben. Die Mittagskost ist meist in Menagen gefüllt und wird an die jeweiligen Gefangenen ausgegeben. Es gibt dabei Normalkost, Krankenkost (medizinisch verordnet), Kostvermehrung (medizinisch verordnet), konfessionsgebundene Kost wie auch für das Frühstück und das Abendessen.

Normalerweise erfolgt die Essensausgabe einzeln im Haftraum, was die Isolation verstärkt. In manchen Vollzugsformen können Gefangene jedoch gemeinsam oder allein in einer eigenen Küche speisen und selbst zubereiten. Ein gemeinsamer Speisesaal, in dem Mahlzeiten an großen Tischen eingenommen und von Inhaftierten gedeckt werden, fördert soziale Interaktionen und trägt zur Sozialisation bei. So entsteht Raum für den Austausch zwischen Menschen verschiedener Kulturen und Glaubensrichtungen, was auch die Identitätsbildung stärkt [56].

Die Einbindung von Ökotrophologen in die Küchenorganisation wäre vorteilhaft, da diese Finanzplanung, Einkauf, Zubereitung und die Einhaltung ernährungsphysiologischer Richtlinien (z. B. DGE) besser überwachen könnten [57]. Eine stärkere Berücksichtigung der Wünsche der Gefangenen bei Ernährung und Darreichung würde soziale Interaktion und Sozialisation zusätzlich fördern.

## Fazit

Strafgefangene und Sicherungsverwahrte sollten in die GKV (SGB V) und die Pflegeversicherung (SGB XI) aufgenommen werden. Modelle, die darauf abzielen, die sicherheitsorientierte Anstaltsmedizin sowie das strukturelle Problem der dualen Loyalitäten [58] und die parallelen Gesundheitsversorgungsstrukturen zu überwinden, setzen sich für eine Neuorganisation der Zuständigkeiten ein. Dabei sollte die Verantwortung vom Justizministerium auf die Gesundheitsministerien übertragen werden, um das Äquivalenzprinzip besser umsetzen zu können. Solche Ansätze werden international derzeit intensiv diskutiert [14] und sollten weiterhin in die Diskussion um die Verbesserung der gesundheitlichen Versorgung in Deutschland einbezogen werden.

Neben Optimierungsmöglichkeiten in den Bereichen Bewegung/Sport und Ernährung ist insbesondere der Zugang zu erprobten, bewährten und anerkannten Hilfe- und Behandlungsmethoden in Haft – im Vergleich zur Situation in Freiheit – in manchen Bereichen (v. a. in der Prävention, der Suchtkrankenversorgung und Infektionsprophylaxe) unzulänglich. Dies kann auf der einen Seite zu Behandlungsdiskontinuitäten führen, mit z. T. erheblichen Auswirkungen auf den gesundheitlichen Status in und nach der Haft (vgl. *Wenner vs. Germany*). Auf der anderen Seite können in Haft z. B. erstmals Diagnostik und Behandlungen von Störungen und Krankheiten stattfinden, die in Freiheit unerkannt bzw. unbehandelt bleiben (z. B. viele somatische Störungen, Hepatitis C und HIV). Entlang des bundesweit einmaligen „Medizinischen Versorgungskonzeptes“ aus Baden-Württemberg [24] sollten folgende Themen intensiver diskutiert und in die Praxis umgesetzt werden:

- Telemedizin in der JVA,
- Sicherstellung einer einheitlichen medizinischen Versorgungsqualität durch die Einrichtung spezialisierter medizinischer Kompetenzzentren,
- Optimierung der medizinischen Versorgung sowie der Teilhabe- und Unterstützungsangebote für körper- und mehrfachbehinderte Gefangene,
- Implementierung einer spezialisierten Schwerstpflegeabteilung in JVK,
- Versorgungsmodelle für ältere und schwerbehinderte Inhaftierte, beispielsweise durch die barrierefreie Gestaltung des Krankenreviers,
- Umstellung auf eine elektronische Krankenakte in JVK,
- Prüfung, ob eine sofortige Vollstreckung der Ersatzfreiheitsstrafe notwendig ist, sowie ggf. der Möglichkeit eines Aufschubs vor der Aufnahme in die JVA,
- Kooperation zwischen Justiz- und Maßregelvollzug auf regionaler sowie landesweiter Ebene mit dem Ziel, eine landesweite Kooperationsvereinbarung zu etablieren,
- Einrichtung von Qualitätszirkeln als zentrales, bereichsübergreifendes Gremium zur Qualitätssicherung und -weiterentwicklung,
- Depotsubstitution und nahtloser Übergang in eine anschließende Substitutionsbehandlung nach der Haftentlassung sowie ein Naloxon-Programm zur Überbrückung im Rahmen des suchttherapeutischen Übergangsmanagements mit dem Ziel der Mortalitätsprophylaxe [59],
- verpflichtender Erwerb der „Fachkunde Suchtmedizin“ für alle Anstaltsärzte,
- Optimierung der Personalressourcen in den Krankenrevieren der JVAen sowie die angemessene personelle Ausstattung der medizinischen Kompetenzzentren,
- Fortbildungsoffensive für Anstaltsärzte und Krankenpflegepersonal,
- Entwicklung und Implementierung von Weiterbildungsprogrammen für Ärzte in der JVA, einschließlich modularer oder abschnittsweiser Qualifizierungen zu spezifischen Facharztkompetenzen. Eine ganzheitliche, interdisziplinäre Sichtweise ist notwendig, die neben der medizinischen Versorgung der Gefangenen auch die Arbeitsbedingungen, den Qualifikationsbedarf und die Gesundheit der Bediensteten sowie die baulichen Rahmenbedingungen der JVA berücksichtigt;
- Ernährung, abgestimmt auf kulturelle Besonderheiten,
- Verbesserte Bewegungs- und Sportangebote.

## Footnotes

1 Vgl. etwa aktuelle Entscheidung des Bundesverfassungsgerichts (2 BvR 1661/23) vom 05.12.2023 zum „Ausschleichen“ aus der Substitutionsbehandlung.

2 Contingency Management bezieht sich auf die Planung und Durchführung von Maßnahmen, die darauf abzielen, Risiken und Herausforderungen in der sozialen und gesundheitlichen Versorgung zu bewältigen [44].
